# Hematoma expansion in intracerebral hemorrhage – the right target?

**DOI:** 10.1186/s42466-023-00256-6

**Published:** 2023-07-27

**Authors:** David Haupenthal, Stefan Schwab, Joji B. Kuramatsu

**Affiliations:** Department of Neurology, University Hospital Erlangen, Friedrich-Alexander-University of Erlangen-Nuremberg (FAU), Schwabachanlage 6, 91054 Erlangen, Germany

**Keywords:** Intracerebral hemorrhage, Hematoma enlargement, Hematoma expansion, Hemostatic therapies, Blood pressure management

## Abstract

**Background:**

The avoidance of hematoma expansion is the most important therapeutic goal during acute care of patients with intracerebral hemorrhage. Hematoma expansion occurs in up to 20–40% of patients and leads to poorer patient outcome in one of the most severe sub-types of stroke.

**Main text:**

At current, randomized controlled trials have failed to provide evidence for interventions that effectively improve functional outcome in patients with intracerebral hemorrhage. Hence, hematoma expansion may serve as important surrogate target that appears causally linked with a poorer prognosis. Therefore, reduction of hematoma expansion rates will eventually translate to improved patient outcome overall. Recent years have shed light on the importance of early and aggressive treatment in order to reduce the risk for hematoma expansion in these patients. Time measures and imaging markers have been identified that may allow patient selection at very high risk for hematoma expansion.

**Conclusions:**

Refinements in patient selection may increase chance for randomized trials to show true benefit. Therefore, this current review article will critically evaluate and discuss available evidence associated with hematoma expansion in patients with intracerebral hemorrhage.

## Introduction

Globally intracerebral hemorrhage (ICH) accounts for up to 27% all acute strokes and is associated with a very high morbidity and mortality [1–3]. Therefore, ICH is an important contributor to the global burden of disease with a loss of disability-adjusted life-years (DALYs) at about 9.5 years for a single event [4]. One of the strongest predictors of functional outcome is initial ICH-volume [5] and therefore hematoma expansion (HE) represents the most important modifiable therapeutic target theoretically translating to improved outcomes [6]. In contrast to ischemic stroke, no randomized controlled trial has proven effect of a single intervention on functional endpoints. Several medical therapeutic approaches such as aggressive systolic blood pressure lowering or hemostatic therapies have so far not succeeded. Hence, more precise patient selection, those at particularly high risk for HE, appears to be an important next step to improve future trial outcomes. The aim of this review is to critically evaluate the existing evidence and to discuss if patient selection could be improved in the future.

## Hematoma expansion and clinical outcomes

Various studies have validated HE as predictor of functional outcome and mortality. The increase of 1ml in absolute ICH-volume leads to a 7% greater likelihood for patients to shift from independence to dependence [7]. A recent study evaluated other mechanisms of injury beyond HE with the loss of DALYs. The loss of DALYs in patients exposed to the respective injury type was greatest in the subgroup with HE as compared to intraventricular hemorrhage or peri-hemorrhagic edema [4]. These results underscore the importance of not only identifying patients at high risk for HE. Yet, they also support the role of HE being interlinked with other important injury mechanisms contributing to poor outcome in ICH.

## Definition of hematoma expansion

Hematoma expansion refers to the growth of a hematoma over time, as observed by serial neuroimaging. The pathophysiology of HE has not been fully understood yet [8]. Several models and biological mechanisms have been suggested as potential factors in the progression of active bleeding and HE. Definitions of clinically significant HE have been inconsistent across publications. Studies have used a dichotomized relative or absolute volume increase as well as a combination of both. Early definitions included a relative volume increase of 33%, 40%, or 50% [9–11]. More recent trials defined HE as relative increase of volume between 33% and/or absolute increase of 6ml to 12.5ml [12, 13]. One study investigated different cut off points (3ml, 6ml or 12.5ml, 26% or 33%) with regard to their sensitivity and specificity for prediction of poor outcome. For each definition the sensitivity was < 50% while the specificity was significantly higher. Furthermore, absolute definitions performed significantly better in predicting severe disability or death. This might be due to the fact that HE is proportional to volume of damaged brain tissue due to its 3-dimensional increase [14].

The HE-rate reported varies widely. Possibly this is related to the heterogeneity of HE-definitions and different time-points of follow-up imaging used for HE assessment. One study analyzing data from 531 patients reported that 13–32% met commonly used HE-definitions [14]. Considering time from symptom onset to diagnostic imaging provided that within the first 3 h any degree of HE was present in up to 73% and clinically significant HE occurred in 35% [7]. Another study (n = 204) reported a progressive decline of HE-rates with increasing time from onset to diagnostic CT [9], i.e. HE occurring in 36% within the first 3 h, in 16% between 3 and 6 h, and in only 6% between 6 and 12 h, with no patient suffering HE between 24 and 48 h.

## Predictors of hematoma expansion

Over time several predictors of HE have been identified and incorporated in trial design.

1) Time from symptom onset to initial imaging.

2) Volume of Intracerebral Hemorrhage.

3) Imaging markers in non-contrast CT and contrast medium CT.

4) Antithrombotic Treatment and Anticoagulation.

## Time from symptom onset to initial imaging

**Fig. 1:**
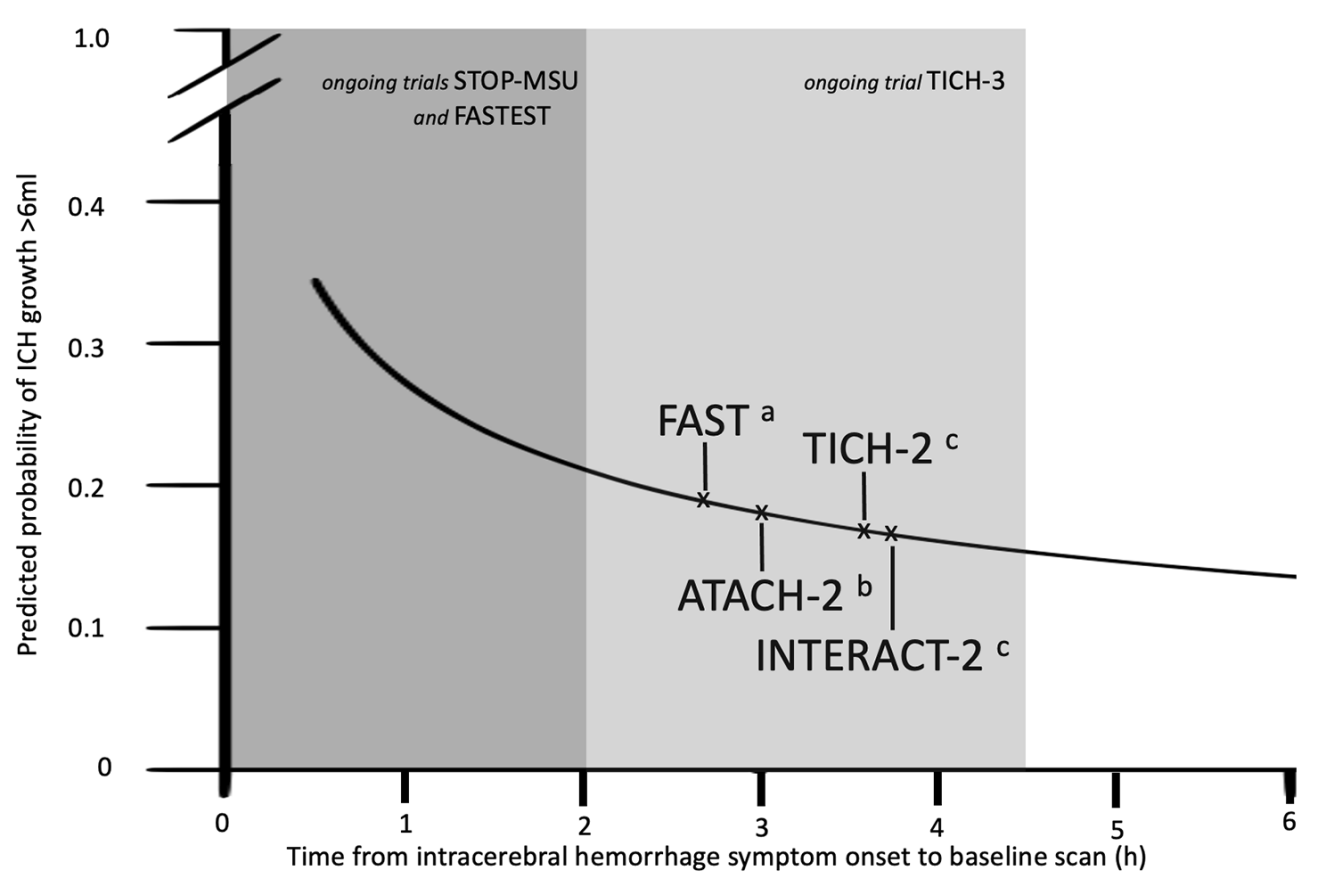
Predicted probability of intracerebral hemorrhage growth > 6ml by time from symptom onset to baseline imaging. Recreated from Al-Shahi Salman et al. [16] Major trials and their respective (**a**) mean time from onset to treatment (**b**) mean time from onset to randomization and (**c**) median time from onset to randomization. Additionally, time window of ongoing trials (shaded)

One important predictor of HE is time to initial diagnostic imaging. A prospective cohort study with over 1000 patients showed that shorter time to initial CT (≤ 6 h vs. >6 h) was an independent predictor of HE (OR 2.55(1.53 to 4.27); p < 0.001) [15]. This was further validated by a large individual patient-data meta-analysis including 5435patients by Salman et al. [16]. Multivariate modeling of HE-predictors showed that the predicted probability for HE declined at increasing time from onset to diagnostic imaging. The decline was steepest between 0.5 and 3 h, indicating that HE is much more probable within the first 3 h. Hence ICH is a dynamic disease (Fig. 1). Thus, one clinically meaningful interpretation might be that the earlier patients reach medical treatment the more likely therapeutic strategies are effective to reduce the risk for HE.

## Volume of intracerebral hemorrhage

**Fig. 2:**
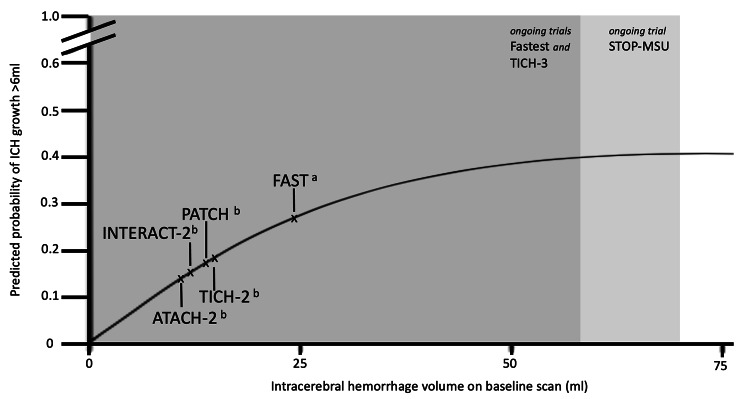
Predicted probability of intracerebral hemorrhage growth > 6ml according to ICH volume on baseline imaging. Recreated from Al-Shahi Salman et al. [16] Major trials and their respective (**a**) mean volume (**b**) median volume. Additionally, eligible ICH volume of ongoing trials (shaded)

**Fig. 3:**
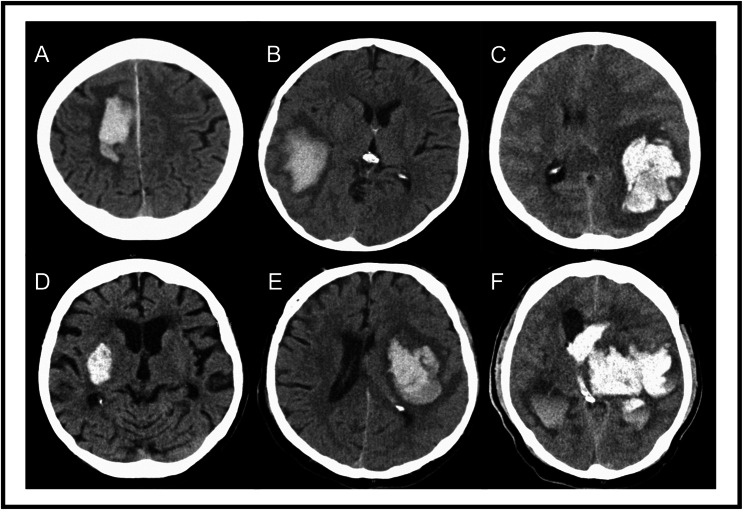
Illustration of different ICH volumes. (**A**) lobar ICH of 10 ml. (**B**) lobar ICH of 30 ml. (**C**) lobar ICH of 60 ml. (**D**) deep ICH of 10 ml. (**E**) deep ICH of 30 ml. (**F**) deep ICH of 60 ml

ICH volume is a well-known predictor of functional outcome and mortality as well as validated predictor of HE itself [5, 17, 18]. A prediction score study (N = 1012) for HE reported that larger ICH volumes of 30-60ml and > 60ml are associated with higher odds for HE than ICH-volumes of 10ml (1.64(1.04.-2.59) and 2.10(1.25–3.55), respectively) [15]. But the question arises, how clinically meaningful (influence of functional outcome) is HE in larger versus small hematomas. A study with pooled individual patient data (n = 1954) showed that odds for unfavorable outcome due to absolute HE is greater in small to medium-sized ICH volumes compared to larger hematomas (OR 3.09(1.52–6.29) versus OR 1.09(0.13–9.20) [19]. This aspect may be related to differences of HE-measurements in absolute versus relative change. For example, the relative change in small ICH with an absolute increase of 3ml is much larger compared to large hematoma volumes. Another question arises, if there is actually a threshold where ICH-volume may not enlarge any further due to the counteracting of the hard skull. In the aforementioned large IPDMA by Salman et al. the predicted probability of HE increased up to a peak at an ICH volume of 75ml after which it declined [16] (Fig. 2, for examples of ICH volume see also Fig. 3).

## Imaging markers in non-contrast CT and contrast medium CT

Neuroimaging has the potential to identify patients with a high likelihood of experiencing HE. Due to its widespread availability CT scans are the primary imaging modality. Both non-contrast and contrast medium CT can be applied in identifying predictors of HE.

Intra-hematomal contrast-medium extravasate on delayed CTA, e.g. spot-sign, has been used to identify patients at increased risk for HE. The prevalence of the spot sign appears to be between 17 and 56% [20]. In a multicenter prospective observational cohort study (n = 268) showed that absolute extent of HE was higher in spot-sign positive patients (8.6ml(-9.3-121.7) vs. 0.4ml (-11.7-98.3)) [21]. Furthermore, mortality at 3 months was higher amongst these patients (43.4% vs. 19.6%; p = 0.001) and functional outcome was worse. Subsequent meta-analyses have confirmed these findings [22–24]. Xu et al. report in a meta-analysis of 29 studies that the spot-sign occurred in about a quarter of patients and was associated with an 8-fold increased risk of HE and a 4-fold increased risk of poor outcome. A comparison of various definitions of the spot-sign in another meta-analysis showed the strongest association with HE and unfavorable functional outcome in a spot-sign, defined as one or more focal areas of contrast pooling of any size and morphology being discontinuous from the normal or abnormal vasculature adjacent to the hemorrhage [23]. In a HE prediction model the addition of the spot-sign to clinical and imaging predictors (e.g. time from onset to baseline imaging, ICH-volume and antiplatelet use) improved the discrimination of that prediction model [16].

**Fig. 4:**
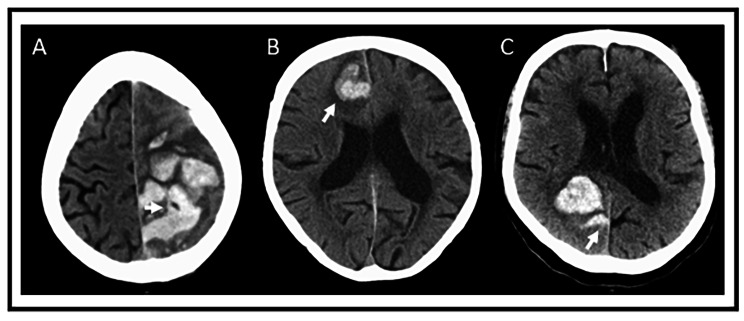
Illustration of different non-contrast CT imaging markers (**A**) Black Hole Sign. (**B**) Blend Sign. (**C**) Island Sign. Images contributed by Prof. A. Dörfler, Director, Institute of Neuroradiology at the University Hospital Erlangen

An alternative to contrast-medium based diagnostics are non-contrast CT markers. They are increasingly investigated regarding their association with HE and functional outcome. In general, these non-contrast CT (NCCT) markers are categorized into density and shape markers. They hypothetically may reflect a hematoma that is not yet stable, which may be reflected by extensions from the hematoma or areas of intra-hematomal hypodensities or fluid-levels. Different signs have been investigated by a very large meta-analysis of 25 studies and 10,650 patients. A variety of markers were found to be associated with higher risk of HE and poor outcome, e.g. black hole sign (OR 3.7(1.42–9.64) and OR 5.26(1.75–15.76), respectively), blend sign (OR 3.49(2.20–5.55) and OR 2.21(1.16–4.18) and island sign (OR 7.87(2.17–28.47) and OR 6.05(4.44–8.24) [25] (also see Fig. 4). Yet, there was relevant heterogeneity and pooled estimates were not adjusted for confounding variables. The use of NCCT markers in risk assessment for HE might seem reasonable, especially in situations or locations with low availability of CTA. Nevertheless, large prospective studies are needed to assess the relevance and specificity over the time course from symptom onset to diagnostic CT. The conclusion arises that technological advances, such as automated artificial intelligence based evaluations need to be urgently developed and validated to advance early imaging based risk-prediction.

## Antithrombotic medication

Patient treated with antiplatelet or anticoagulant drugs are at a higher and prolonged risk of HE. While several studies, e.g. The Cerebral Hemorrhage and NXY-059 Treamtent (CHANT) trail, could not find an association between antiplatelet therapy (ATP) and HE [26–28], other retrospective studies and a meta-analysis documented that ATP is an independent predictor of HE. A retrospective study among 251 patients found an OR of 7.67 (1.62–36.4) for a HE of 40% on the second hospital day [29]. In the only meta-analysis with seven cohorts and 1465 patients the OR for HE under APT was 1.85 (1.37–2.50) [30].

The prior use of vitamin K antagonists is associated with a higher rate of HE. In a small prospective study among 70 patients Flibotte et al. found an OR of 6.2 (1.69–22.88) for a HE, defined as an increase of greater 33%. They also noted that HE was found later, suggesting a prolonged bleeding [26]. Further evidence is found in the CHANT trail providing an OR of 3.49 (1.27–9.55) [28]. In a larger prospective cohort (n = 510) the OR was higher in lobar ICH (4.44 (1.19–17.63)) than in deep ICH 2.08 (1.20–3.61) [31].

Regarding non-vitamin K antagonists (NOAC) a large multicenter observational cohort study among 1328 patients with oral-anticoagulation related ICH could not find a significant difference in rate of HE between NOAC and VKA (33.6% vs. 34.2%; p = 0.89) [32]. Nevertheless, there was a higher rate of HE among NOAC-ICH patients with relevant anticoagulatory activity compared to those without such activity (38.9% vs. 16.7%; p = 0.03).

1. Hemostatic therapies
2. BP Management

The rationale to use hemostatic agents in patients with acute spontaneous ICH to prevent HE is at hand. This might even more apply to patients with known prior use of antithrombotic medications, such as antiplatelet agents or oral anticoagulation. Various agents with different modes of action have been investigated by randomized trials.

## Recombinant factor VIIa

The rFVIIa (recombinant factor VIIa) is established in the therapy of diseases such as congenital or acquired hemophilia. It is an important initiator of hemostasis. The factor VII tissue complex activates the conversion of factor X to Xa by forming a complex with exposed tissue factors of the ruptured vessels. Thereby it is activating the hemostatic pathway locally at the region of endothelial disruption [8]. After smaller pilot trials, the first larger trial was a phase 2b dose-finding trial (n = 399) in 2005 to investigate the influence of rFVIIa on HE in patients with ICH [33]. Patients within 3 h after symptom onset were assigned to either receive placebo or 40, 80 or 160 µg rFVIIa per kilogram of body weight. HE was higher in the placebo group compared to the rFVIIa groups (absolute mean increase 8.7(4.9–12.4) vs. 4.2(2.0-6.3); p = 0.01). Severe disability or death (mRS 4–6) was also higher in the placebo group (69% vs. 53%); p = 0.004). Yet there was a non-significant difference in the rates of thromboembolic serious adverse events in disfavor of the treatment group (7% vs. 2%; p = 0.12). When administered early within 3 h after onset, the treatment effect of rFVIIa was even greater (absolute increase in ICH volume 10.7ml in the placebo group vs. 4.4ml in the treatment group; p = 0.009). Following these promising results, the phase 3 Recombinant Factor VIIa in Acute Intracerebral Hemorrhage Trial (FAST) randomly assigned 841 patients to receive placebo (n = 268) or one of two doses (20 or 80 µg) of rFVIIa (n = 276 and n = 297, respectively) [34]. In-line with the previous study, expansion of ICH-volumes was lower among the treatment groups compared with placebo (2.6ml (-0.3-5.5); p = 0.08 in the 20 µg group; 3.8ml (0.9–6.7; p = 0.009) in the 80 µg group). Yet, this difference did not translate into improved outcomes, defined as mRS 5 to 6, (24% in the placebo group, 26% in the group receiving 20 µg of rFVIIa per kilogram, and 29% in the group receiving 80 µg). In addition to that, arterial TAE occurred more often in the in the group receiving 80 µg of rFVIIa than in the placebo group (9% vs. 4%; p = 0.04). A post hoc analysis aimed to identify the subgroup of patients who would benefit from the therapy [35]. Patients ≤ 70 years with baseline ICH volume < 60mL, IVH volume < 5mL, and time from onset-to-treatment ≤ 2.5 h had a significantly lower OR for poor outcome, defined as mRS 5 to 6, (OR 0.28 (0.08–1.06)). A meta-analysis consisting of 5 RCTs confirmed the findings from the FAST trial [36]. While rFVIIa reduces hematoma growth, it failed to improve survival or functional outcome after ICH. Furthermore, the incidence of TAE was higher (OR 1.56(0.98–2.48)).

The ongoing rFVIIa for Acute Hemorrhagic Stroke Administered at Earliest Time (FASTEST, NCT03496883) trial has now incorporated post-hoc findings into its study protocol. It is attempting to apply the treatment approach to a sub-group within an early time window of 120 min after stroke onset. This global phase 3 randomized, double-blind controlled trial will compare a rFVIIa 80 µg/kg dose plus best standard therapy to placebo and best standard therapy alone. An estimated number of 860 patients with ICH and without prior use of oral anticoagulants, aged ≥ 18 and ≤ 80, will be included with an ICH volume of ≥ 2 and < 60ml, smaller volumes of intraventricular hemorrhage (IVH score ≤ 7), Glasgow Coma Scale of ≥ 8, and treatment initiated within 120 min from stroke onset. Half of the patients are expected to be treated within the first 90 min. The primary outcome will be the ordinal mRS (0–3 vs. 4–6) at 180 days. The trial started recruiting in December 2021 and is expected to be completed by December 2025.

## Platelet transfusions

Following several observational studies, the Platelet transfusion versus standard care after acute stroke due to spontaneous cerebral hemorrhage associated with antiplatelet therapy (PATCH) trial was the first and to date only randomized trial to investigate platelet transfusion in ICH-patients with antiplatelet therapy [37]. A relatively modest number of 190 patients with ICH and known prior use of antiplatelet medication, enrolled within 6 h after symptom onset, were assigned to either receive platelet transfusion or standard care. The results showed that the odds for a shift towards death or dependence at 3 months were higher in the platelet transfusion group than in standard care group (adjusted common OR 2.05(1.18–3.56; p = 0.011). There was a similar distribution of mRS estimates among patients receiving platelet transfusion within the first 3 h and 3 to 6 h. Yet, a greater proportion of patients died during hospital stay as compared to standard treatment (23 (24%) vs. 16 (17%)). There were imbalances between the transfusion arm and control arm. Median ICH volume was 13.1ml and 8.0ml in the platelet transfusion group and standard care group, respectively. Median HE volumes at 24 h were not significantly different between groups (2.01ml vs. 1.16ml; p = 0.81). It is debated whether the absolute increase associated with antiplatelet therapy was too small to be sufficiently influenced by this therapy. Furthermore, patients receiving platelet transfusion were more likely to experience any SAE. One of the possible reasons for the negative trial result, beside statistical imbalances, might be that platelet transfusion may have resulted in a stimulation of proinflammatory prothrombotic effects. Therefore, the current American Heart Association guideline recommends against platelet transfusions in general and may only be considered for emergency craniotomy for hematoma removal [38].

## Tranexamic acid

Tranexamic acid, which is an effective hemostatic agent and antifibrinolytic drug, has been used in disease entities [39]. In a large RCT with 20,211 trauma patients, it has been shown to significantly reduce mortality [40]. In a subsequent subgroup analysis of traumatic ICH patients there was both a non-significant reduction of mean hemorrhage growth (-3.8ml (-11.5-3.9); p = 0.33) and death (aOR 0.47(0.21 to 1.04)) among patients treated with tranexamic acid compared to placebo [41]. In 2014 the feasibility of a RCT of tranexamic acid in spontaneous ICH was proven in a small study with 24 patients enrolled [42]. Following this, the Tranexamic acid for hyperacute primary IntraCerebral Haemorrhage (TICH-2) phase 3 superiority study was conducted with 2325 ICH patients enrolled between 2013 and 2017 [13]. Within 8 h of symptom onset patients either received 1 g intravenous tranexamic acid over 10 min followed by an 8 h infusion or matching placebo. Regarding its primary outcome of mRS at day 90 the study failed to achieve a significant difference between the two treatment groups (aOR 0.88(0.76–1.03); p = 0.11). Yet, volume of HE at 24 h was smaller among patients treated with tranexamic acid (mean difference − 1,37 (-2,71–0.04); p = 0.04) and the rate of significant HE, defined as increase of ≥6ml or growth of ≥33%, was significantly smaller among these patients (265 (25%) vs. 304 (29%); p = 0.03). It should be noted that TXA resulted in lower mortality by day 7 (aOR 0.73(0.53–0.99), p = 0.041) and fewer SAEs at 90 days (521 [45%] vs. 556 [48%], p = 0.039). The authors state that the small reduction of HE might have been not sufficient enough to result in an improved functional status. Furthermore, the median time from onset to randomization was 3.6 h (2.6–5.1) and only 36% of patients were randomized within the first 3 h in the treatment group. The most recent meta-analysis of seven trials and involving 2650 patients TXA was found to reduce HE significantly on follow-up imaging (OR 0.825(0.692–0.984); p = 0.033) compared to placebo [43]. Importantly, this effect was time dependend. It was found only in patients treated within 4.5 h of symptom onset (OR 0.823 (0.690–0.980); p = 0.029) but not among patients treated later than 4.5 h (OR 1.026(0.795–1.324); p = 0.844).

Currently, there are three trials investigating treatment effects of TXA in ICH patients. The Treatment of Intracerebral Hemorrhage in Patients on Non-vitamin K Antagonist (TICH-NOAC, NCT02866838) is investigating the treatment effect of TXA in Xa-Inhibitor associated ICH. Selection criteria comprise patients within 12 h of symptom onset and without restrictions regarding ICH-volume. Enrollment of 64 patients has been completed but results have not been published yet. Nevertheless, conference reports provide negative results on outcome and HE. The phase II double-blind, randomized, placebo-controlled, multicenter Stopping intracerebral haemorrhage with tranexamic acid for hyperacute onset presentation including mobile stroke units (STOP-MSU, NCT03385928) trial is an ongoing trial investigating the effects of treatment with TXA or placebo within 2 h of symptom onset. Patients (n = 326) will be randomized in a 1:1 ratio to either receive TXA or placebo. Only patients with an ICH volume < 70ml can be included. The primary efficacy measure is the proportion of patients with relevant HE, defined as relative increase of hematoma volume of ≥33% or ≥6 ml absolute increase after 24 h. Completion of this trial is estimated to be in March 2024. The phase III, prospective, randomized, placebo-controlled, multicenter Tranexamic acid for IntraCerebral Haemorrhage 3 (TICH-3, ISRCTN97695350) trial is another ongoing trial assessing the treatment effect of TXA or placebo in ICH. A comparatively large number of Patients (n = 5500) with an ICH volume of < 60ml will be included. The time window is limited to a maximum of 4.5 h after symptom onset instead of max. 8 h as in TICH-2 according to the aforementioned results of the meta-analysis by Gut et al. The primary outcome is mortality at seven days and secondary functional outcome is mRS at day 180. The trial is scheduled to be completed in February 2028.

## Anticoagulation associated ICH

As aforementioned patients treated with oral anticoagulation are at the highest risk for HE with HE rates up to 40%. Therefore, specific hemostatic treatment appears logical and paramount in those patients to limit HE. Yet, large sized RCT are missing and at current most data is derived from large observational or prospective studies investigating specific antidotes. A small trial investigating hemostatic treatment in vitamin-K-antagonist ICH, the International Normalized Ratio Normalization in Coumadin Associated Intracerebral Hemorrhage (INCH) trial [44], compared fresh frozen plasma (FFP) against prothrombin complex concentrate (PCC). This randomized, open-label, blinded-endpoint trial included patients presenting within 12 h after symptom onset and an INR of at least 2.0. The trial was stopped early after inclusion of 50 patients after a safety analysis. The primary endpoint of INR ≤ 1.2 within 3 h of treatment was achieved in 2 (9%) patients in the FFP group and in 16 (67%) in the PCC group (aOR 30.6 (4.7-197.9); p = 0.0003). The HE after 24 h in the PCC group was 16.4ml (2.9–29.9) less than among patients treated with FFP (p = 0.018). Of note, five of eight deaths in the FFP group were related to HE compared to none of five deaths in the PCC group. There was no significant difference between both groups regarding functional outcome at 3 months. But the trial was not designed for this endpoint. Two agents have been approved for antagonizing NOAC-associated hemorrhages: Idarucizumab in dabigatran-related ICH and andexanet alfa in factor Xa inhibitor-related ICH. However, to date there is little data available regarding effect on HE-rates or clinical endpoints, as both studies did not specifically address patients with ICH. Idarucizumab can be used for specific reversal of dabigatran. The non-competitive inhibitor is a humanized monoclonal antibody fragment binding to dabigatran with high affinity [45]. Although it has been approved by the Federal Drug Administration and European Medicines Agency in 2015 for reversal of dabigatran associated life-threating bleeding complications or for patients requiring emergency surgery, data in patients with ICH and potential effects on HE in dabigatran-related ICH is merely lacking. An observational study reported HE in 3/27 (11.1%) ICH patients on follow up CT scans, which appears smaller than expected in anticoagulation associated ICH [46]. Few studies compared andexanet alpha to PCC treatment. A registry analysis of patients with 3030 Xa-Inhibitor-related hospitalizations for major bleeds by ICD codes found a lower mortality rate among andexanet alpha treated patients (6/67(9.0%) vs. 43/170(25.3%)) [47]. But the study did not provide information on hematoma characteristics or functional outcome. The most robust study to date, pooled data from the multicenter prospective single-arm ANNEXA4-trial with Data from a multicenter observational cohort study (RETRACE-II) [48]. Among 182 patients included in this study (85 receiving andexanet alpha and 97 receiving standard of care, i.e. prothrombin complex concentrates). Patients treated with Andexanet alpha exhibited lower rates of HE, defined as increase of ≥ 35%, (11/80 (14%) vs. 21/67 (36%); p = 0.002) with an absolute difference of ICH-volume − 8.21 (-12.93—3.5); p < 0.001). Yet again, the prevention of HE did not translate into an improved functional outcome. Rates of in-hospital mortality (14/85 (16.5%) vs. 20/97 (20.6%); p = 0.48) and proportion of patients with mRS 4 to 6 were lower in the andexanet alpha group (64.3% vs. 70.6%; p = 0.4), but these differences did not reach statistical significance. To evaluate the efficacy and safety of andexanet alpha compared to usual care in fXi-ICH the prospective, randomized open-label, phase 4 ANNEXA-I trial is currently conducted (NCT03661528). Patients will be randomized within 6 h after symptom onset to either receive andexanet alpha or any treatment that is considered appropriate by the treating physicians. The primary outcome measure is the rate of effective hemostasis, defined as change from baseline NIHSS + 6 or less after 12 h and ≤ 35% increase of hematoma volume at 12 h and no rescue therapies administered between 3 and 12 h after randomization. 1200 patients are expected to be enrolled by July 2024.

## Blood pressure management

Initial systolic blood pressure (SBP) of > 200mmHg, known as acute hypertensive response in ICH, is an independent predictor of HE and increased mortality [49]. Considering that HE occurs predominantly within the first hours, aggressive systolic blood pressure lowering seems to represent a logical and promising approach to improve patient outcome. Consequently, several large prospective RCTs investigated this treatment approach.

## INTERACT-Trials

In a first pilot trial Intensive blood pressure reduction in acute cerebral hemorrhage (INTERACT-1) Anderson et al. investigated the safety and efficiency of lowering elevated blood pressure after acute ICH [50]. Patients with acute ICH and a SBP of 150-220mmHg in at least two measurements were included. Patients were randomly divided into two treatment groups: intensive lowering of blood pressure (target SBP 140mmHg; n = 203) and standard guideline-based management of BP (target SBP 180mmHg; n = 201) within 1 h and maintenance over 7d or until discharge. There was significant difference regarding mean proportional hematoma growth at 24 h between the guideline group and the intensive group (difference 22.6% (0.6–44.5); p = 0.04). Yet, this difference failed to reach significance after adjustment for initial hematoma volume and time from onset to CT (16% vs. 7%; p = 0.06). In the intensive group there was a smaller but non-significant substantial hematoma growth (Difference 8% (-1.0-17.0%)) and mean absolute increase in hematoma volume (Difference 1.7ml (-0.5-3.9) after 24 h. Among patients enrolled within the first 4 h from onset there was a substantial reduction in the rate of hematoma growth in favor of the intensive group (15% vs. 30%). Intensive BP lowering did not result an increase of SAE at day 90. The main-phase study INTERACT-2 used a similar trial design [51]. 2839 patients were randomly assigned. In contrast to INTERACT-1, the primary outcome was death or major disability, defined as mRS score 3 to 6 at 90d. The target blood pressure of < 140mmHg within the first hour was only achieved in 33.4% in the intensive-treatment group. The primary outcome was not significantly reduced by aggressive BP-lowering (52% vs. 55.6%; p = 0.06) but prespecified ordinal shift-analysis showed benefit in favor of the intensive treatment group. In contradiction to the findings from INTERACT-1 aggressive BP lowering did not result in a significant decrease of absolute or proportional decrease of HE (absolute decrease 1.8ml(-0.3-3.8); p = 0.091; relative decrease 7.5 (-31.9-47.0); p = 0.708). Regarding secondary outcomes, including all-cause mortality, rebleeding and safety outcomes, no significant differences were found. It is worth noting that 41% of the patients were randomized ≥4 h after symptom onset. Furthermore, patients with large ICH were excluded based on investigator judgement without providing a clear cut-off.

## ATACH-II

The second large phase 3 trial investigating the impact of intensive blood pressure lowering is the Antihypertensive Treatment of Acute Cerebral Hemorrhage-2 (ATACH-2) trial [52]. It was a multinational multicenter RCT enrolling patients between 2010 and 2015. The target SBP throughout the 24 h after randomization in the intervention group was 110 to 139mmHg and in the control group 140 to 179mmHg, respectively. Initially patients could be enrolled within the first 3 h after onset. As HE is the primary target of BP lowering, the time window was expanded to 4.5 h because studies showed a comparable prevalence of HE within the first 3 h and 3 h to 4.5 h. The primary outcome was moderate to severe disability or death, defined as a mRS of 4–6, at 3 months. Enrollment was discontinued for futility early after 1000 patients enrolled out of 1280 originally planned, as pre-specified interim analyses showed an absolute difference regarding the primary outcome of only 1% between both groups (rate of disability or death at 90d 38.7 vs. 37.3%, OR 1.04(0.85–1.27); p = 0.72). Although the rate of HE was lower in the intensive group, it failed to reach statistical significance (19% vs. 24%; p = 0.09). The rate of renal adverse events within the first 7d were higher among patients in the intensive-treatment group (9% vs. 4%; p = 0.002) and the rate of SAE after 3 months tended to be higher in this group as well (25.6%vs. 20.0%; p = 0.06). Over recent years, a number of posthoc- and meta-analyses of the major RCTs have been conducted [53]. Findings throughout these analyses are that intensive blood-pressure management appears to be safe and the rate of HE is lower with intensive blood pressure treatment. Achieved SBP reductions were associated with functional status (improvement per 10mmHg increase adjusted OR 0.90(0.87–0.94]; p < 0.0001). Notably, a post hoc analysis of the ATACH-2 trial found a higher rate of patients with good outcome among patients in the intensive treatment group when treated within the first 2 h after symptom onset (OR 1.68 (1.01–2.83); p = 0.048) [54]. One explanation might be that blood pressure reduction is time-dependent and therefore, time to antihypertensive treatment is of importance. The currently ongoing Intensive Ambulance-delivered Blood Pressure Reduction in Hyper-Acute Stroke Trial (INTERACT4) is investigating the effect of pre-hospital blood pressure reduction on functional outcome and HE (NCT03790800).

## Discussion

To date randomized controlled trials targeting acute treatment interventions in patients with primary ICH have failed to provide robust benefit. Controversy exists if hematoma enlargement is the right target at all [55]. Nevertheless, HE is an easily quantifiable biomarker, assessment using CT scans has very good accuracy, and pathophysiological causality on clinical outcomes is at hand. What may be the reasons for negative trial results: (1) HE may just be the wrong target to impact long-term outcomes, (2) the amount of HE reduction is too small to translate into improved functional outcome (Table 1), (3) the proportion of patients at high risk for HE within trials was too small, (4) the intervention itself leads to adverse events or secondary complications influencing endpoints.

**Table 1:** Major Clinical Trials targeting hematoma expansion with provided Time from onset to randomization

Major Trial	Time from onset to randomization	ICH Volume (ml)	HE (abs. ml)
FAST	Intervention: 161 ±37, min^a^ Control: 160 ±38, min^a^	I: 24 ± 26 C: 23 ± 26	I: 4.9 (2.9-7.0) C: 7.5 (5.4–9.6)
PATCH	Intervention: n.a. Control: n.a.	I: 13.1 (5.4–42.4) C: 8.0 (4.4–25.8)	I: 2.01 (0.32–9.34) C:1.16 (0.03–4.42)
TICH-2	I: 3.6 (2.6–5.1), h C: 3.7 (2.6-5.0), h	I: 14.1 (5.9–32.4) C: 12.5 (5.1 to 31.9)	I: 3.72 (±15.9) C: 4.9 (±16)
INTERACT-2	I: 3.7 (2.8 to 4.8), h C: 3.7 (2.9 to 4.7), h	I: 11 (6–19) C: 11(6–20)	I: 3.1 (2.1–4.1) C: 4.9 (3.1–6.6)
ATACH-2	I: 182.2 (57.2), min C: 184.7 (56.7), min	I: 10.2 (2.3–85.2) C: 10.2 (0.98–79.1)	I: n.a. C: n.a.

ICH Volume and Hematoma enlargement. ^a^ Time from onset to treatment.

Hematoma expansion is validated predictor of outcome in ICH patients. Considering that, it appears less likely that HE is the wrong target to be aimed at. However, the two largest trials in ICH patients (e.g. INTERACT-2, TICH-2) showed a trend towards improved outcomes, one trial for blood pressure management and the other on hemostatic treatment. Therefore, it seems apparent that optimizing patient selection could lead to benefit, yet limiting generalizability (see Table 2). Statistically the greater the proportion of patients with high risk for HE in the trial population is, the larger the potential benefit of the intervention could be. Thus in simple terms, approaches aiming to prevent HE to the greatest extent possible should be combined.

**Table 2:** List of approaches for improvement of future ICH-trials

Approaches for improvement of future trials
- Improving selection criteria: o Limitation of max. eligible time from symptom onset to randomization/intervention (< 4.5 h) o Limitation of max. eligible ICH-volume on initial imaging (< 60ml) o Stratify for antithrombotic medication o Stratification of ICH locations o Radiological predictors (non-contrast and contrast medium imaging markers o Development of AI-based risk prediction models
- Standardizing the definition of HE (stratified according to ICH location)
- Endpoint selection: o Functional outcome assessment at ≥6 months o Shift analysis models for HE and outcome instead of dichotomization o Patient-oriented continuous outcome variable (e.g. QALYs) o Composite endpoints of radiological and clinical endpoints

Results of the meta-analysis by Salman et al. offers important insights [16]. The two most important clinical selection criteria are timing since symptom onset and ICH volume. Treatment should be performed as early as possible, as the risk of HE is highest within the first three hours after onset of bleeding. Major trials such as INTERACT-2 and ATACH-2 had median times from onset to randomization greater than three hours. A post-hoc analyses of the FAST and ATACH-2 trial identified the “early” subgroup that would benefit most from interventions. This, in turn, contributed to the design of the FASTEST trial, in which patients are assigned to the intervention within the first 120 min. The previous large trials may have had a too large time window until randomization and start of the intervention, respectively. If the risk for HE has already decreased significantly in a large proportion of patients, the remaining study population with a theoretical chance of benefit, will be too small to translate to benefit for the entire study population (Fig. 1). But only including patients within the first two hours may potentially lead to difficulties in recruitment and limits generalizability of effects. In addition, prior use of antithrombotic medications may modify these time windows, whereas in patients with anticoagulation-associated ICH, the time window may be kept wider, as patients exhibit HE at longer durations, especially if hemostasis is not achieved. Nevertheless, it should be kept in mind that reducing the time window will exclude those patients with larger or unknown time windows. In one study, the proportion of patients with a time window > 3 h was 56.5% and those with an unknown time window was 25.6% [56]. While patients with ischemic stroke in an unknown time window can sometimes be assigned to therapy based on multimodality imaging, these would be missing for ICH trials. These aspects pronounce the importance of imaging predictors to overcome those limitations.

The other important aspect is ICH volume, as higher baseline ICH volumes are associated with a greater risk for HE but only up to a certain volume threshold. Therefore, ICH volume stratification does make sense from a statistical perspective [6]. The predicted probability for HE plateaus at an ICH volume of about 75ml (Fig. 2). Yet, mortality in patients with ICH volumes > 60ml ranges between 71% and 93% according to hematoma location [5]. Hence, the question may be raised to which extent prevention of a few milliliters of rebleeding actually influences functional outcome in these patients with very large ICH. In particular, this is true when the outcome is recorded dichotomously. On the other hand, small hemorrhages have a lower risk of rebleeding and the effect of an intervention in terms of preventing HE may be reduced. Consequently, the above mentioned currently recruiting trials incorporated volume limits (FASTEST: 2-60ml, ANNEXA-I: 0.5-60ml, STOP-MSU < 70ml).

Clinical inclusion criteria should be extended to include radiological criteria. Due to the high availability, low risk for patients and the positive association between non-contrast CT and contrast-medium CT with HE, imaging markers should be thoroughly evaluated for potential use in future trials in order to identify patients at high risk for HE. Ideally, potential imaging biomarker should not only be a predictor of HE but also be an independent predictor of the functional outcome itself. If, in addition, there were an association with the time of symptom onset, it might also be possible to include patients with an unknown time window.

In the future, blood biomarkers could be investigated for their potential use in trial design for HE and ICH. Biomarkers such as matrix metalloproteinase 9 or interleukin 6 have been shown to be associated with HE [57]. Yet, extended further research is needed to validate these blood biomarkers regarding their utility in optimizing patient selection, ideally implemented by point of care devices.

Further, within future trials meticulous care should be taken that the control arm may not be influenced by prior data, e.g. ATACH-2 trial which was stopped for futility, possibly due to the fact that blood pressure lowering within the control arm was almost identical to the intervention arm of the INTERACT-2 trial. Contrary, it should be kept in mind that narrowing inclusion criteria lead to a decrease in generalizability of the results. Therefore, in more inclusive cohorts these aspects should be considered in prespecified subgroup analyses to assess the heterogeneity of treatment effects.

Not only patient selection but also endpoint selection represents a possible starting point. Frequently used radiological endpoints are dichotomized as absolute or relative HE, but dichotomization always leads to loss of information. To date, neither absolute nor relative ICH volume growth has been superior, and therefore, a combination of the two is used as an endpoint is most trials. In small baseline hematomas even a small increase of absolute volume results in a greater relative growth, while the clinical impact on functional outcome might be perceived as being relatively low. But this may also diverge in different ICH locations (e.g. deep ICH) and is strongly related to the eloquence of the region affected, i.e. in deep ICH within a region of high eloquence minor changes in hematoma volume lead to a larger deterioration of functional outcome [6]. Choosing the right cut points is as important as difficult. The ideal cut point should have a high probability that a patient, meeting the definition, has a higher likelihood of poor outcome [14]. Yet, it should be kept in mind that a higher cut point may result in an increased specificity regarding clinical impact, but on the other hand exhibits has a lower sensitivity [14]. Hence, shift analysis models also for HE could be valuable [58], as shown convincingly for functional endpoints in stroke. Furthermore, the question has been raised whether the mRS is sensitive enough to detect small differences as outcome parameters and whether patient-oriented outcome measures (e.g. QALYs or cognitive assessment) might be more appropriate. Using continuous outcome variables could allow to statistically measure even small treatment effects in the future trials. However, such analyses are currently still limited in ICH [59]. In addition, perhaps a later assessment of functional outcome, e.g. at 6 or 12 month, would better capture the full potential of rehabilitation after ICH and appropriate intervention.

Attempts have been made to combine radiological and clinical endpoints, e.g. HE at 24 h and concurrent symptomatic worsening or HE at 24 h and poor outcome at 30d or later. These composite outcomes may potentially lead to greater chance for trial success, yet may rather obscure true treatment effects on outcome that matters to the patient and may limit interpretability regarding causal inference [6].

## Conclusion

At current, randomized controlled trials have failed to provide evidence for interventions that effectively improve functional outcome in patients with intracerebral hemorrhage. In future trials clinical and radiographic inclusion criteria should be chosen carefully to improve patient selection. The most important clinical selection criteria appears to be timing since symptom onset to prevent hematoma expansion, hence, time is brain not only in ischemic stroke!

## Data Availability

Data sharing not applicable to this article as no datasets were generated or analyzed during the current study.

## List of abbreviations

DALY: Disability-adjusted life-year
HE: Hematoma expansion
ICH: Intracerebral hemorrhage
IPDMA: Individual patient data meta-analysis
IVH: Intraventricular hemorrhage
mRS: Modified Rankin Scale
n.a.: Not available
NCCT: Non-contrast CT
OR: Odds ratio
PHE: Peri-hemorrhagic edema
QALY: Quality-adjusted life-year
RCT: Randomized controlled trial
rFVIIa: Recombinant factor VIIa
SBP: Systolic blood pressure
SD: Standard deviation
TAE: Thrombembolic serious adverse event
TXA: Tranexamic acid
